# Molecular pathogenesis, mechanism and therapy of Cav1 in prostate cancer

**DOI:** 10.1007/s12672-023-00813-0

**Published:** 2023-11-01

**Authors:** Qiang Bian, Bei Li, Luting Zhang, Yinuo Sun, Zhankui Zhao, Yi Ding, Honglian Yu

**Affiliations:** 1 Department of Pathophysiology, Weifang Medicine University, Weifang, 261053 Shandong People’s Republic of China; 2 Department of Biochemistry, Jining Medical University, Jining, 272067 Shandong People’s Republic of China; 3 The Affiliated Hospital of Jining Medical University, Jining Medical University, Jining, 272100 Shandong People’s Republic of China; 4 Department of Radiological Image, Zhengzhou University People’s Hospital, Zhengzhou, 450003 Henan People’s Republic of China

**Keywords:** Prostate cancer, Cav1, Pathogenesis, Mechanism, Treatment

## Abstract

Prostate cancer is the second incidence of malignant tumors in men worldwide. Its incidence and mortality are increasing year by year. Enhanced expression of Cav1 in prostate cancer has been linked to both proliferation and metastasis of cancer cells, influencing disease progression. Dysregulation of the Cav1 gene shows a notable association with prostate cancer. Nevertheless, there is no systematic review to report about molecular signal mechanism of Cav1 and drug treatment in prostate cancer. This article reviews the structure, physiological and pathological functions of Cav1, the pathogenic signaling pathways involved in prostate cancer, and the current drug treatment of prostate cancer. Cav1 mainly affects the occurrence of prostate cancer through AKT/mTOR, H-RAS/PLCε, CD147/MMPs and other pathways, as well as substance metabolism including lipid metabolism and aerobic glycolysis. Baicalein, simvastatin, triptolide and other drugs can effectively inhibit the growth of prostate cancer. As a biomarker of prostate cancer, Cav1 may provide a potential therapeutic target for the treatment of prostate cancer.

## Introduction

Prostate cancer (PCa) is the most common malignant tumor of the male urinary system. It mainly affects men over 60 years of age. In recent years, as living standards elevate and population aging intensifies, the incidence of PCa has experienced a marked surge, casting a profound impact on the quality of human life [1, 2]. Contemporary radical prostatectomy is predominantly used for treatment paradigms of initial-stage PCa. For more advanced, locally invasive, or metastatic cases of PCa, therapeutic strategies typically pivot towards chemotherapy and androgen-deprivation therapy [3–6]. To date, the pathogenesis and underlying molecular biology of PCa are inadequately elucidated [7]. Further research into the pathogenesis of PCa is needed to find useful therapeutic approaches.

Cavelin-1 (Cav1) is located on the long arm of chromosome 7, and is localized at the D7S522 locus (7q31.1), which is a fragile site that is easily lost in cancer [8]. It has been found that deletion of the q31 region of chromosome 7 is closely related to PCa progression and death [9]. A large number of literatures have shown that Cav1 is closely related to PCa, which affect the occurrence and development of PCa [3, 10, 11]. Some scholars have found that the main function of secreted Cav1 in the microenvironment of PCa is to promote angiogenesis and cell survival [12]. In PCa, the abnormally expressed Cav1 interacts with AKT and activates specific oncogenic activities, leading to faster progression of PCa, which is not conducive to the treatment of PCa. Drugs such as baicalin [13], simvastatin [14], triptolide [15], and various chemicals such as phenylbutyrate [16], incadronate [17], cholesterol and phytosterols [18] affect Cav1 expression or participate in certain signaling pathways that delay the progression of PCa. Although a large number of drugs have been used to treat PCa, the therapeutic effect of some drugs has decreased year by year, leading to an increase in the recurrence rate of PCa. The mechanism of some drugs needs to require further study.

In this review, we introduce the function and structure of Cav1 and its relationship with clinical parameters, explore the signaling pathways involved in PCa, and how pharmacological regulation of Cav1 expression affects the progression of PCa. This will allow us to further understand the role of Cav1 in PCa and find more therapeutic methods for PCa.

## Structure and function of Cav1

Caveolin represents a distinct vesicular structure present on the cellular membrane surface, serving as an integral structural and functional protein [19]. The caveolin family comprises three subtypes, including Cav1, Cav2, and Cav3 [20, 21]. Cav1 and Cav2 are widely expressed in normal human cells and tissues. Cav1 exhibits prevalent expression within a diverse array of cell types, including but not limited to vascular endothelial cells, adipose tissue, smooth muscle cells, and stromal cells. Cav3 is specifically expressed in muscle and has been reported in skeletal muscle tissue and cardiomyocytes [22, 23]. The structure and function of Cav1 are further elaborated.

**Fig. 1:**
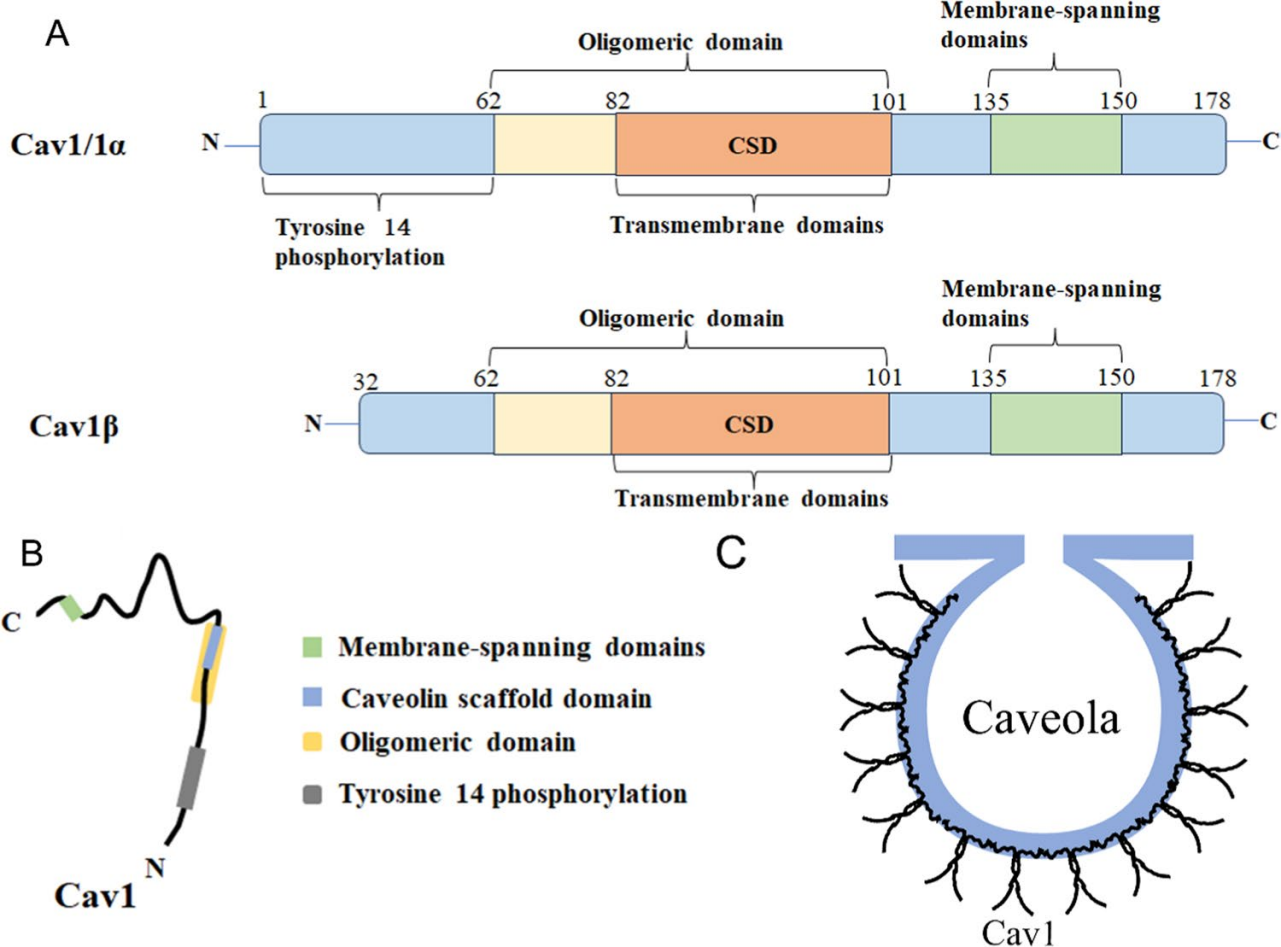
Structure diagram of Cav1. **A** The structure of Cav1 mainly includes Tyrosine 14 phosphorylation domain, oligomeric domain, CSD and membrane-spanning domains. The CSD is located between amino acids 82–101. **B** The polarized structure of Cav1. **C** Oligomerization of Cav1 monomers forms multimeric vesicles

Cav1 is a structural membrane protein with a molecular weight of approximately 21 to 24 kDa. Cav1 is an essential component of the globular invagination of most types of plasma membranes and functions as a scaffold protein for caveolae (Fig. 1C). It is mainly composed of three exons located at 7q31.1 [24], with both N and C terminal facing the cytoplasm. Unlike Cav2 and Cav3, Cav1 has several domains, including tyrosine 14 phosphorylation (residues 1–60) [25], the N-terminal oligomeric domain (residues 61–101) [26, 27], the caveolin scaffold domain (CSD) (residues 82–101) [28], as well as transmembrane domains (residues 82–101) and membrane-spanning domains (residues 135–150) [29] (Fig. 1A). Tyrosine 14 phosphorylation is closely related to the migration and invasion of tumor cells [30]. The residues 61–101 have homologous oligomerization activity and fusion with GST proteins results in GST multimerization [27]. The CSD has the ability to participate in and regulate protein–protein interactions, and it also plays a crucial role in orchestrating the extensive signaling events requisite for endothelial cell recruitment and tumor progression [31]. The polarized structure of Cav1 is also shown [32, 33] (Fig. 1B). Cav1 contains two variants, cav1α (residues 1 to 178) and cav1β (residues 34 to 178) (Fig. 1A), which can be generated by alternative initiation of the same transcript or by alternative transcripts [34–36]. Cav1 phosphorylation is thought to be associated with cell migration and metastasis [37]. There are still other domains that need further investigation.

### Physiological functions of Cav1

Cav1 expression in different cells and tissues can produce different physiological functions. Cav1 promotes adipogenesis when secreted by adipose tissue and adipocytes [38]. Cav1 has tumor suppressive and radioresistant effects if expressed in interstitial fibroblasts [33, 39]. Cav1 has been found in mitochondria and caveolin, but it is usually not expressed in the prostate epithelium or is rarely expressed. It is also involved in a variety of cellular processes such as molecular transport, cell transport, cell adhesion, signaling transduction between molecules, cell cycle change, cell endocytosis [20, 40–43]. Cav1 is also a type II AR coregulator that regulates some cholesterol and steroid receptors, such as estrogen receptors [25]. Cav1 can be secreted into the extracellular environment, which is an exogenous Cav1 and has a promoting effect on the formation of prostate balls. Cav1 regulates the proliferation and differentiation of vascular endothelial cells and is associated with a variety of physiological functions [44–46]. When Cav1 is silenced in stromal cells, the production of cholesterol and testosterone are increase [11].

### Pathological function of Cav1

A large number of literature studies have shown that Cav1 is involved in various processes of tumorigenesis, progression, migration and invasion [13, 47, 48]. It has been reported that Cav1 belongs to an anti-apoptotic protein and is related to bcl family [49–51]. Cav1 is present in different cell types, tumor stages and locations, and its biological effects may vary. Cav1 may be closely related to the progression of some malignant tumors, such as bladder cancer [52, 53], ovarian cancer [54, 55], lung cancer [56, 57], among which its role in PCa [58, 59] being the most extensively explored. Cav1 expression is different in different types and stages of PCa, which affects the progression of PCa.

### Relationship between Cav1 Expression and PCa

In PCa, the expression of Cav1 is correlated with clinical stage, pathological characteristics and other clinical parameters. Some studies have found that the Cav1 expression and metastases [14, 60], gleason score [11, 61–72], survival rate [10, 11, 14, 64, 69–71, 73, 74], clinical stage [10, 72–74] and lymph node involvement [68, 70, 75] are correlated. Cav1 expression is not correlated with age [14, 41, 69–71, 74–77]. The relationship between Cav1 and prostate specific antigen (PSA) is controversial, some studies suggest that there is a significant correlation between Cav1 and PSA in castration-resistant prostate cancer (CRPC) [61, 63, 64, 71, 72, 76], but some studies suggest that there is no significant correlation between Cav1 and PSA of difference stage and grade of PCa [14, 41, 69, 70, 73, 74] (Table 1). The relationship between Cav1 and PSA needs to be further studied.

**Table 1:** Relationship between Cav1 expression and clinical parameters in PCa

Specimen	Sample size	Methods	Gleason	Survival rate	Age	Grade	Other outcomes	Correlation degree	Refs.
Plasma	112/150	PCR	+	unk	unk	unk	PSA	+	[61]
Plasma	80 (40/40)	IHC		+	+	unk	tPSA	−	[73]
							Smoking habits	−	
		PCR					BMI	−	
							Clinical stage	+	
Plasma	542	ELISA	+	unk	+	unk	Clinical stage	−	[62]
Plasma	82/15	WB	+	unk	unk	+			[63]
		ELISA							
Plasma	58(36/22)	ELISA	+	unk	−	unk	PSA	+	[75]
		WB							
		qRT-PCR					T grade	+	
							Lymph node involvement	+	
Plasma	232	IHC	+	+	unk	unk	PSA	+	[64]
Plasma	419	ELISA	−	+	−	unk	PSA	−	[74]
							Lymph node involvement	−	
Tissue	47/20	IHC	unk	unk	unk	+	Clinical stage	+	[19]
							PSA	−	
Tissue	18/18	IHC	+	unk	unk	unk			[65]
Tissue	129	PCR	unk	unk	−	unk	PSA	+	[76]
Tissue	69	RT-qPCR	+	unk	unk	unk	PT stage	+	[66]
Tissue	104	IHC	+	unk	unk	unk			[67]
Tissue	61	WB	unk	unk	unk	unk	Metastases	+	[60]
		IHC							
Tissue	395	qRT-PCR	+		unk	unk	Tumor stage	+	[68]
		IHC							
Tissue	189	IHC	+	+	−	unk	Lymph node involvement	+	[69]
							Positive surgical margins	+	
							PSA	−	
Tissue	71/71	IHC	−	unk	−	−			[77]
Tissue	3117	IHC	+	+	−	unk	PSA	−	[70]
							Lymph node involvement	+	
Plasma/tissue	20/40	qRT-PCR	+	+	−	−	PSA	+	[71]
Tissue	724	qRT-PCR	+	+	unk	unk	Clinical stage	+	[11]
		Flow cytometry							
Plasma/tissue	70/56 45/36	IHC	unk	+	−	unk	PSA	−	[14]
		ELISA							
		RT-qPCR					Metastases	+	
		WB							
Tissue	134/86	WB	−	unk	−	unk	PSA	−	[41]
		PCR (Taq-PCR)							
Plasma	58 (36/22)	qRT-PCR	+	unk	−	unk	PSA	+	[72]
							Clinical stage	+	
Cell	3	qPCR	unk	+	unk	unk			[10]
		ELISA							

^*^[unk] = unknown, [ +] = positive effect, [-] = no effect

## Role of Cav1 in PCa

Cav1 is secreted by PCa cells and observed in the serum of PCa patients, showing a trend of positive correlation with tumor stage and grade. Cav1 is an autocrine/paracrine factor that is up-regulated in metastatic PCa, CRPC and androgen insensitive prostate cancer, but not in hormone sensitive prostate cancer [75]. When Cav1 is overexpressed, it will lead to the proliferation of PCa [78, 79]. Cav1 is associated with ID-1 (differentiation and DNA binding inhibitors) in the helix-loop-helix transcription factor family via HLH structure and has implications for EMT and cell survival in PCa [80]. Non-caveolar Cav1 influences the expression of VEGF-A in PCa cells, thereby impacting the proliferation, migration, and invasion of lymphatic endothelial cells. These alterations, in turn, bear significant implications for the prognosis and survival rates associated with PCa. It has been shown that the expression level of Cav1 in stromal is negatively correlated with epithelial Cav1 expression and AKT activation, affecting the metastasis and invasion of PCa.

The regulation of testosterone and various growth factors in PCa patients can affect the expression of Cav1. Testosterone can affect the survival and growth of PCa cells by regulating [81]. Cav1 can regulate androgen-insensitive prostate cancer cells. It has been reported that inhibition of Cav1 expression can change the insensitivity of androgen-insensitive prostate cancer cells to androgen and slow down the progression of PCa [82]. C-myc belongs to an oncogene, it is closely related to PCa, c-myc induces apoptosis of PCa cells, but the increased expression of Cav1 leads to the decreased apoptotic ability of PCa cells [67]. Cav1 can regulate the expression of acetyl-CoA carboxylase-1 (ACC1) and fatty acid synthase (FASN) in the adipose tissue of PCa cells, participate in the synthesis of fatty acid, further regulate the hormone resistance, and affect the progression and resistance of PCa.

## Cav1 and signal pathway in PCa

**Fig. 2:**
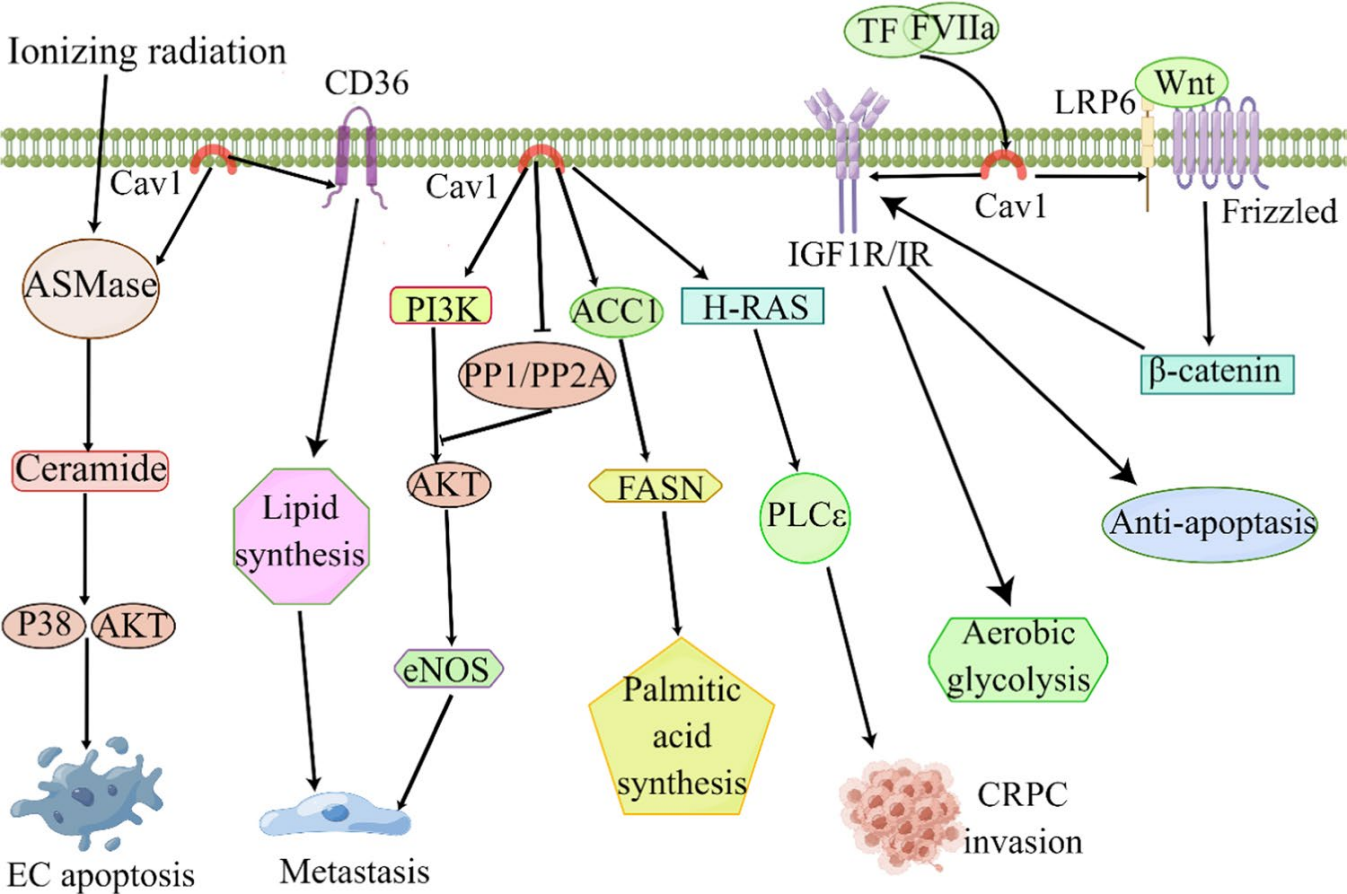
Molecular signaling pathways involved in Cav1 in prostate cancer. Cav1 can promote the apoptosis, migration and invasion of prostate cancer through ASMase, PI3K and H-RAS signaling pathways. Cav1 is also involved in lipid synthesis, palmitic acid synthesis, and aerobic glycolysis

Cav1 plays a pivotal role in an array of signaling pathways. It encompasses a compact 20-amino acid domain, which allows for numerous signaling molecules to interact with Cav1 within the caveolin structure [83]. They include protein kinase C isoforms, heterotrimeric G protein subunits, GTP enzymes, endothelial nitric oxide synthase, SRC-associated tyrosine kinases, epidermal growth factor receptor (EGF-R), phospholipase Cγ1 (PLCγ1), integrin β1 (ITGβ1), p53 and integrin-related proteins [32, 34, 84–86]. The progression of PCa is affected by multiple signaling pathways. Therefore, we will review the signal pathways of Cav1 involved in PCa (Fig. 2).

### Wnt-β-catenin

Wnt proteins are important components of a family of cysteine-rich secretory ligands that affect growth and development in nematodes and mammals [87]. Wnt-β-catenin can regulate cell proliferation and differentiation, and even lead to tumor formation. Cav1 can activate the Wnt-β-catenin pathway and induce internalization of low-density lipoprotein receptor-associated protein 6 (LRP6) [88]. Within the context of PCa, Cav1 assumes a pivotal role in lipid anabolism, particularly in the sphere of lipid synthesis. The regulation of lipid metabolism is an important factor in tumorigenesis. Cav1 can regulate the phosphorylation of LRP6 in PCa, and the interaction between them affects the Wnt-β-Catenin signaling pathway, which in turn affects the phosphorylation of IGF-1R/IR, activates the insulin/IGF-1R pathway, and indirectly affects aerobic glycolysis [89]. Therefore, altering Cav1 expression in human body can affect the lipid anabolic process mediated by Wnt-β-catenin signaling pathway, thereby slowing the progression of PCa.

### PI3K-AKT-eNOS

In PCa, Cav1 fosters cell migration, tubule formation, and nitric oxide production by modulating the PI3K-AKT-eNOS signaling pathway. [90]. Cav1 is able to stimulate angiogenic activity mainly due to CSD mediated. The CSD is where many signaling proteins interact [91]. The expression of Cav1 increases vascular endothelial growth factor by activating AKT phosphorylation, which promotes the proliferation of endothelial cells and is of great significance for angiogenesis [92, 93]. PP1 and PP2A belong to serine/threonine protein phosphatases, and they are closely associated with the phosphorylation of AKT and eNOS [94]. Cav1 overexpression inhibits PP1/PP2A expression, leading to phosphorylation of AKT and eNOS [95]. The combined effect of eNOS and NO affects the migration ability of PCa [96]. Cav1 can stimulate the angiogenesis of PCa through the P13K-AKT-eNOS signaling pathway, which leads to PCa more likely to metastasize.

### ACC1-FASN

In PCa, Cav1 expression was significantly positively correlated with ACC1 and FASN expression, further indicating that Cav1 is involved in the process of fatty acid synthesis [97]. The effects of CD36 and ACC1/FASN are similar. CD36 belongs to the fatty acid translocase, which transports cholesterol and long-chain fatty acids across the plasma membrane. Cav1 interacts with CD36 and affects lipid synthesis, thereby affecting tumor metastasis [90]. Cav1 can interact with ACC1 and FASN to affect the growth of PCa cells. FASN and ACC1 are closely related to fatty acid synthesis [98, 99]. FASN belongs to the relatively large homodimeric enzymes [100]. It produces long-chain fatty acids. Cav1 can alter the RNA expression of ACC1 and FASN and promote palmitic acid synthesis, and FASN/Cav1 interacts to make Cav1 palmitoylated [101]. The signal pathway was supported by that overexpression of Cav1 in PCa promotes ACC1-FASN expression, leading to lipid synthesis and further promoting hormone resistance [102], which can lead to decreased efficacy of hormone therapy and affect the survival of PCa.

### Acid sphingomyelinase (ASMase)/ceramide

The acid sphingosine hydrolase (ASMase)/ Ceramide pathway plays a crucial role in the radiation resistance of cancer cells and radiation-induced endothelial cell (EC) apoptosis. ASMase is highly expressed in EC, which may indicate that EC is sensitive to radiation-induced apoptosis [103, 104]. Cav1 regulates the ASMase/Ceramide mediated ionizing radiation response [105]. Ionizing radiation has some effect on the raft microdomain [106, 107] of cytoplasmic membrane organization. High-dose ionizing radiation can increase membrane ceramide content through the ASMase-Ceramide pathway. When Cav1 is deficient in endothelial cells, membrane signalers are increased, which can affect the downstream targets P38 and AKT [108] and ultimately lead to EC apoptosis. Thus, Cav1 can regulate ceramide-dependent plasma membrane organization. Ceramide-dependent plasma membrane architecture can in turn influence the radiation response of EC and adjacent PCa cells, so as to play a role in the treatment of PCa.

### TF/FVIIa/IGF1R

Tissue factor (TF) is a transmembrane protein and a promoter of blood coagulation. TF is able to bind factor VII (FVII) and FVIIa to form a TF/FVIIa complex [109]. IGF-1R belongs to the transmembrane receptor tyrosine kinase [110], which mainly regulates cell proliferation, apoptosis and migration. IGF-1 binds to IGF-1 receptor (IGF-1R) and IR to activate the migration pathway of PCa, which may lead to the lethal development of PCa. Both TF and IGF-1R are able to bind CSD [111]. The CSD of Cav1 regulates FVIIa-induced phosphorylation of IGF-1R. TF/FVIIa induces the phosphorylation of Cav1 tyrosine 14 via β1 integrin. In PCa, Cav1 and β1 integrins play a role in the anti-apoptotic signaling of TF/FVIIa/IGF1R [109].

### H-RAS/ PLCε

Phosphatidylinositol-specific phospholipase Cε (PLCε) is a member of the human phosphatidylinositol-PLC family. In contrast to other phospholipase Cε isoforms, PLCε has a domain of a GTPase nucleotide exchange factor that can be used to activate Ras family GTPases, but it also is regulated by RAS family GTP [112]. RAS is the most frequently mutated gene family in cancer, and H-RAS belongs to RAS family. PLCε expression is increased in urologic tumors, and it promotes AR nuclear translocation in PCa [113]. The expression of H-RAS and PLCε is positively correlated with Cav1. PLCε regulates CRPC invasion and migration in metastatic CRPC. When Cav1 expression is reduced, it can reduce PLCε expression through H-RAS, thereby inhibiting CRPC invasion and migration [14]. Further studies on Cav1 inhibitors targeting the Cav1/H-RAS/PLCε pathway is needed to slow the progression of CRPC.

## Targeting Cav1 signaling pathway in PCa therapy

So far, a large number of drugs such as baicalein, simvastatin, triptolide and some chemicals have been used to treat PCa. These drugs mainly affect some signaling pathways involved in Cav1 to treat PCa (Table 2). We further explored the specific effects of these drugs.

**Table 2:** Effect of drugs on Cav1 expression in PCa

Drug	Target	Role	Refs
Baicalein	Cav1/AKT/mTOR	Inhibition of prostate cancer cells migration and growth	[13]
Simvastatin	Cav1/H-RAS/PLCε	Reduce castration-resistant prostate cancer metastasis and androgen receptor antagonist resistance	[14]
Triptolide	Cav1/CD147/MMPs	Inhibit the proliferation, migration and invasion of prostate cancer cells	[15]
ProstaCaid	G2/M phase	Inhibition of adhesion, migration and invasion of prostate cancer cells	[114]
dasatinib	RTK/TK	Inhibit the proliferation of prostate cancer cells	[115]
sunitinib			
Phenylbutyrate	Histone deacetylases	Blocking the invasive ability of prostate cancer cells	[16]
Incadronate	isoprenoid biosynthesis pathway	Inhibition of prostate cancer cells migration	[17]

### Baicalein

Scutellaria is an herb and baicalein can be extracted from the root of Scutellaria. Baicalein is a bioactive flavonoid that has been shown to treat hypertension, infectious diseases, inflammation, and cardiovascular diseases [116]. Most importantly, baicalein has anti-tumor effects on cancer cells [117], and it is able to inhibit the anti-apoptotic ability of Cav1 [118]. Baicalein inhibited the activation and phosphorylation of AKT. The downstream product of AKT is mTOR, and phosphorylated mTOR is able to promote cancer cell proliferation, migration, and invasion [119]. The anticancer effect of baicalein was enhanced when AKT expression was inhibited. Therefore, baicalein may inhibit the occurrence and development of androgen-independent prostate cancer through the Cav1/AKT/mTOR pathway [13]. Baicalein can still be used as a drug for the treatment of androgen-independent prostate cancer and improve the survival rate.

### Simvastatin

Simvastatin is an inhibitor of Hydroxymethylglutaryl Coenzyme A (HMG-CoA) reductase, which plays a role in the conversion of HMG-CoA to mevalonate and prevents cholesterol biosynthesis [120]. Cav1 is involved in cholesterol regulation, and it can directly bind to cholesterol to form caveolin [121]. Cav1 is highly expressed in metastatic CRPC. Simvastatin can regulate the expression of Cav1 and inhibit the migration of CRPC through H-RAS/PLCε pathway. It has been shown that simvastatin inhibits de novo cholesterol synthesis in CRPC cells and inhibits Cav-1 expression, which further enhances the anticancer effect of androgen receptor antagonists [14]. Inhibition of Cav1 expression or cholesterol synthesis by simvastatin combined with some drugs may still be a therapeutic strategy for CRPC.

### Triptolide

Triptolide is mainly extracted from a medicinal plant, tripterygium wilfordii, which is a diterpene trioxide. It has the functions of resisting inflammation, immunosuppression and promoting apoptosis. Triptolide can inhibit the migration and invasion of PCa cells through the Cav1/CD147/MMPs pathway [15]. CD147, a member of the immunoglobulin superfamily, plays a critical role in numerous processes including the secretion of matrix metalloproteinases (MMPs), intercellular communication, and the invasion and migration of tumor cells [122, 123]. CD147 exhibits a high degree of specificity in binding to the 39-amino acid domains of Cav1. This interaction with MMPs exerts a significant influence on the process of tumor invasion [124]. Triptolide can down-regulate the expression of Cav-1, CD147 and MMPs to inhibit the migration and invasion of PCa cells. In the future, the combination of Cav1 inhibitor and triptolide may more effective treatment of aggressive PCa.

### Dasatinib and sunitinib

Dasatinib, an oral agent capable of activating the Bcr-Abl enzyme and inhibiting the Src family kinase (SFK) protein [125, 126], inhibits the proliferation and migration of PCa cells. Sunitinib (SU11248) operates as a receptor tyrosine kinase (RTK) inhibitor that targets vascular endothelial growth factor (VEGF), exhibiting both antiangiogenic and antitumor properties [127, 128]. In PCa, Cav1 is overexpressed, leading to PCa cells proliferation and migration. Studies have shown that dasatinib and sunitinib affect Cav1 expression by inhibiting RTK/TK signaling activity in PCa cells. Dasatinib and sunitinib combined with anti-Cav1 antibody can significantly inhibit the proliferation and migration of PCa cells [115]. This will provide us with a new way to combine drugs with anti-Cav1 antibodies in the treatment of PCa.

### Other chemical reagents

Phenylbutyrate (PB), characterized as a short-chain fatty acid, acts through the inhibition of histone deacetylase activity [16]. It is capable of inducing the differentiation and apoptosis of PCa cells [129, 130]. It has been found that PB can inhibit the expression of Cav1 in PCa cell, leading to a decrease in the invasive ability of PCa cells. Incadronate is a third-generation bisphosphonate. It inhibits the expression of Cav1 by inhibiting key enzymes in isoprenoid biosynthesis pathway and affects the progression of PCa [17]. ProstaCaid (PC) is a dietary supplement consisting of 33 Chinese herbs and nutrients, including ganoderma lucidum, resveratrol, epigallocatechin-3-gallate, vitamin D3 [114], etc. Vitamin D3 can down-regulate Cav1 expression, inhibit MMP-9 activity, and affect cell cycle changes. The common feature of these drugs is their ability to affect Cav1 expression, which in turn hinders the progression of PCa.

## Conclusion

In this review, structure, the signaling pathways and therapeutic drugs involved in Cav1 are summarized, mainly to provide theoretical basis for better treatment of PCa. Its unstable location and CSD domain make Cav1 play a critical role in PCa. Cav1 indirectly affects lipid anabolic processes in Wnt-β-catenin pathway and glycolytic pathway through insulin/IGF-IR pathway. Cav1 can affect tumor angiogenesis through PI3K-AKT-eNOS. The activation of ACC1-FASN pathway leads to hormone resistance of PCa cells and is not conducive to hormone therapy of PCa. Cav1 affects the expression of ceramide in the ASMase/ceramide pathway, thereby affecting the sensitivity of PCa cells to radiotherapy and reducing the effect of radiotherapy. The TF/FVIIa/IGF1R pathway has an anti-apoptotic effect, and TF/FVIIa activates Cav1 and β-1 integrin and affects the expression of IGF1R. These signaling pathways are not conducive to the treatment and prognosis of PCa. Therapeutic agents such as baicalin, simvastatin, triptolide, prostacaid, dasatinib, and sunitinib have been leveraged in PCa treatment, influencing the signaling pathways implicated in Cav1 regulation. In order to improve the survival rate of PCa patients, the pathway of Cav1 involved in PCa still needs to be further studied.

## Future perspectives

Taken together, Cav1 serves a crucial function in the molecular mechanism of PCa. The role of Cav1 in modulating multiple signaling pathways in PCa holds substantial significance for therapeutic strategies. ACC1-FASN, Tf/FVIIa/IGF-1R/IR and AKT signal pathways might be potential pathways in the treatment of PCa. The utilization of novel nanotechnology to combine chemical drugs like phenylbutyric acid and incadronate, or anti-Cav1 antibody, with PCa drugs such as baicalein and triptolide, holds potential for a new direction in PCa treatment. More research could be focused on the molecular mechanism of Cav1 and drug therapy in PCa.

## Data Availability

Not applicable.
